# A fully automatic MRI‐guided decision support system for lumbar disc herniation using machine learning

**DOI:** 10.1002/jsp2.1342

**Published:** 2024-05-30

**Authors:** Di Zhang, Jiawei Du, Jiaxiao Shi, Yundong Zhang, Siyue Jia, Xingyu Liu, Yu Wu, Yicheng An, Shibo Zhu, Dayu Pan, Wei Zhang, Yiling Zhang, Shiqing Feng

**Affiliations:** ^1^ Department of Orthopaedics Tianjin Medical University General Hospital Tianjin People's Republic of China; ^2^ Beijing Longwood Valley Company Beijing People's Republic of China; ^3^ School of Control Science and Engineering, Shandong University Jinan People's Republic of China

**Keywords:** artificial intelligence, diagnosis, lumbar disc herniation, MSU classification, Pfirrmann grade

## Abstract

**Background:**

Normalized decision support system for lumbar disc herniation (LDH) will improve reproducibility compared with subjective clinical diagnosis and treatment. Magnetic resonance imaging (MRI) plays an essential role in the evaluation of LDH. This study aimed to develop an MRI‐based decision support system for LDH, which evaluates lumbar discs in a reproducible, consistent, and reliable manner.

**Methods:**

The research team proposed a system based on machine learning that was trained and tested by a large, manually labeled data set comprising 217 patients' MRI scans (3255 lumbar discs). The system analyzes the radiological features of identified discs to diagnose herniation and classifies discs by Pfirrmann grade and MSU classification. Based on the assessment, the system provides clinical advice.

**Results:**

Eventually, the accuracy of the diagnosis process reached 95.83%. An 83.5% agreement was observed between the system's prediction and the ground‐truth in the Pfirrmann grade. In the case of MSU classification, 95.0% precision was achieved. With the assistance of this system, the accuracy, interpretation efficiency and interrater agreement among surgeons were improved substantially.

**Conclusion:**

This system showed considerable accuracy and efficiency, and therefore could serve as an objective reference for the diagnosis and treatment procedure in clinical practice.

## Graphical abstract

This study presents the development of a machine learning‐based magnetic resonance imaging decision support system aimed at improving the diagnosis and treatment planning for lumbar disc herniation (LDH). It utilizes a large data set to analyze radiological features and classify lumbar discs, achieving high accuracy in diagnosis and interrater reliability among surgeons. The system offers a reproducible and objective approach to assist clinical decision‐making in LDH management.

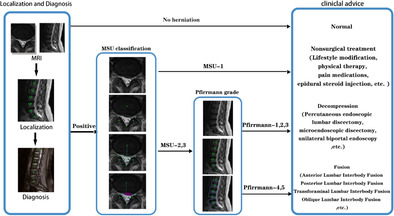

**Abbreviations**
AI: artificial intelligence
CNN: convolution neural network
CT: computed tomography
DSC: dice similarity coefficient
Faster‐RCNN: Faster region‐convolution neural network
HPI: histogram of pixel intensity
IOU: intersection over union
IVD: intervertebral disc
LBP: local binary pattern
LDH: lumbar disc herniation
ML: machine learning
MLP: multi‐layer perceptron
MRI: magnetic resonance imaging
MSE: mean squared error
NLP: natural language process
PHOG: Pyramid Histogram of Oriented Gradients
ROI: region of interest
RPN: region proposal network
TTA: test time augmentation

## 1 INTRODUCTION

Lumbar disc herniation (LDH) is the leading cause of spinal pain worldwide. Statistically, individuals with LDH comprise a significant portion of the global disabled population.^1^, ^2^ It has been estimated that LDH affects nearly 2.8 million people annually in the United States.^3^

Radiographic evidence is an indispensable basis for surgeons to choose the appropriate treatment. Magnetic resonance imaging (MRI) is the most reliable tool to assess the state of intervertebral discs (IVDs) due to its advantage of soft‐tissue contrast, noninvasive, radiation‐free, and appropriate representation of IVD changes.^4^, ^5^, ^6^

Composed of annulus fibrosus and nucleus pulposus, IVD always serves as a shock absorber between the adjacent vertebrates. Due to age‐related degeneration, annulus fibrosus will wear out and cause the leakage of the nucleus pulposus, which could lead to direct compression and inflammation. That is to say, the most critical aspect in the evaluation of LDH is the degeneration condition of IVD, size and location of the herniation. The MSU classification and Pfirrmann grade are famous and well‐tested tools for the evaluation of IVD conditions.^6^, ^7^, ^8^, ^9^, ^10^, ^11^ The MSU classification could describe the size and location of the herniated disc by elaborative region partition.^7^ In the meantime, Pfirrmann grade could quantify the degeneration degree of IVD objectively.^12^ Both the MSU classification and Pfirrmann grade have been proven to hold prognostic value for surgical selection.^13^, ^14^

Manual analysis of MRI images could lead to subjective variability. Additionally, a set of MRI images often comprises hundreds of individual images. Evaluating herniated discs is a tedious and time‐consuming task in clinical practice. Therefore, automating this process would be beneficial.

The application of machine learning (ML) in medical imaging has seen significant advancements in recent years. These technologies, harnessing algorithms such as convolutional neural networks and support vector machines, have revolutionized the way medical images are analyzed, offering enhanced accuracy and efficiency.^15^, ^16^ Particularly in spinal imaging, ML models have demonstrated their potential in automating the diagnosis and evaluation processes, which are traditionally labor‐intensive and prone to human error.^17^, ^18^, ^19^, ^20^, ^21^, ^22^

To date, while various ML models have been developed to classify disc degeneration, none have integrated the multifaceted aspects of IVD assessment—encompassing the size, location, and degree of degeneration—into a single, unified model.^23^, ^24^, ^25^, ^26^ Our approach aims to fill this gap by providing a comprehensive assessment tool. Such a model could provide the surgeon with an accurate and reliable tool to diagnose LDH and evaluate IVD. As a result, better treatment options would be selected, and the efficiency of outpatient as well as inpatient departments would be improved. In this study, an ML model was developed to automatically diagnose and provide treatment advice based on Pfirrmann grading and MSU classification. Once the ML model was trained, the performance was evaluated with a crossover study.

## 2 METHODS

### 2.1 Systemic overview

**FIGURE 1:**
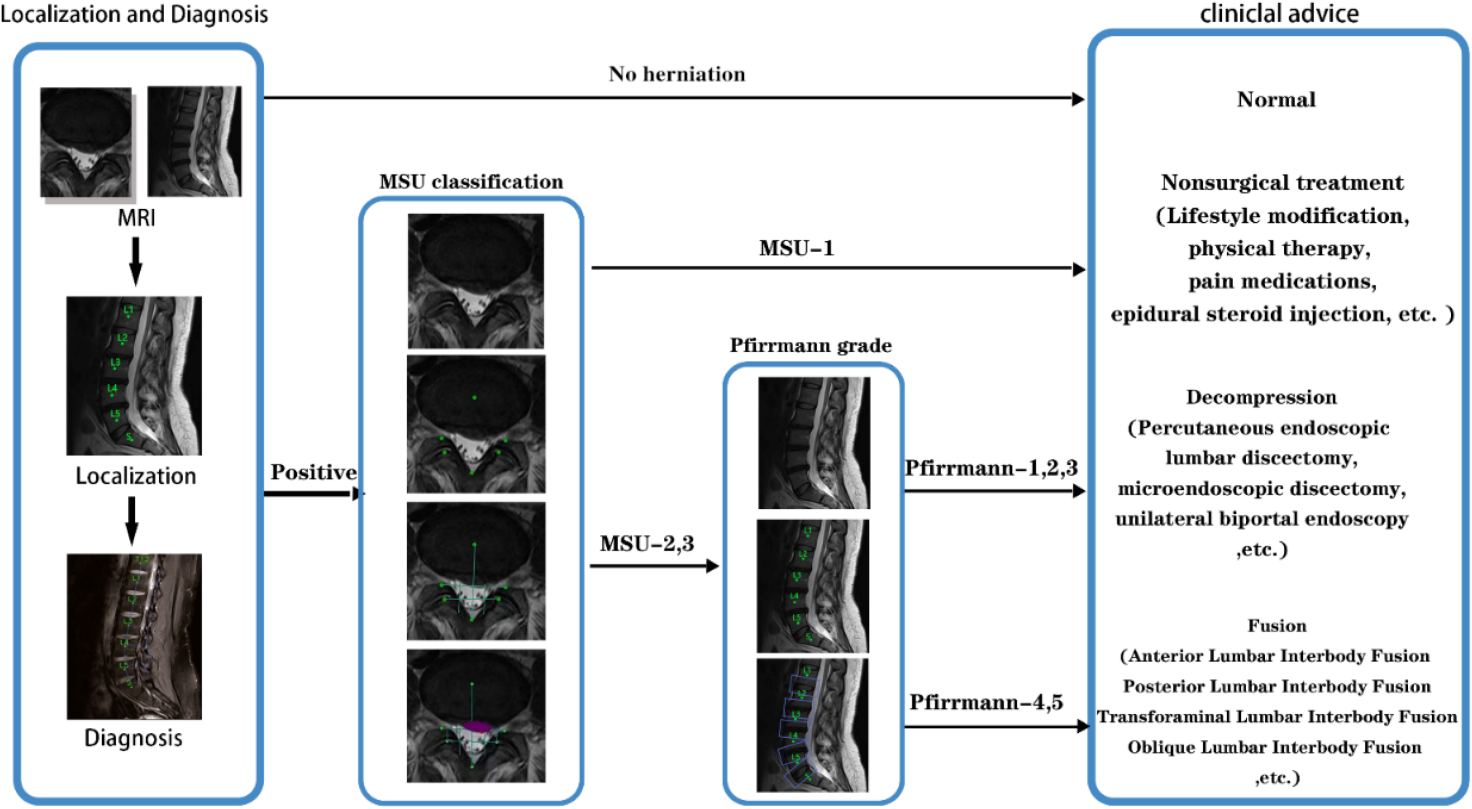
The flowchart of the system.

The research team proposed a clinical decision‐making system based on T2‐weighted MRI images using ML. In the system, the first step was the localization of the IVD on the MRI sagittal view. Then, the system analyzed the radiological features of identified discs in the sagittal view to diagnose LDH. Subsequently, if a disc was evaluated as herniated in the sagittal view, the cross‐section view of MRI images would be correlated and analyzed. In the cross‐section view, the first step was to localize the marker points defining the region of MSU classification. Subsequently, the herniated area will be segmented out. Then the MSU classification will be made based on the localization of the herniated area. After that Pfirrmann grade would be assessed for MSU‐2,3 discs and clinical advice would be provided. The flowchart of the system is shown in Figure 1.

### 2.2 Image preprocessing and annotation

This study excluded the poor‐quality images and obscured the related personal information using Synedra View Professional. The images were resized into 512*512 pixels using OpenCV. The training and test data sets were labeled by five board‐certified orthopedic surgeons (J.X.S. with 6 years of experience, D.Z. with 10 years of experience, Y.W. with 6 years of experience, J.W.D. with 3 years of experience, and S.Y.J. with 5 years of experience). The images would be reviewed by S.Q.F if there was a divergence.

For the localization task, we employed image augmentation techniques such as minor rotations, flips, the addition of a small amount of Gaussian noise, and slight contrast adjustments to increase the model's robustness to variations in image quality. For other tasks, including diagnosis, MSU classification, and Pfirrmann grading, augmentation was limited to minor rotations and flips to maintain the critical diagnostic features of the images.

### 2.3 IVD localization

**FIGURE 2:**
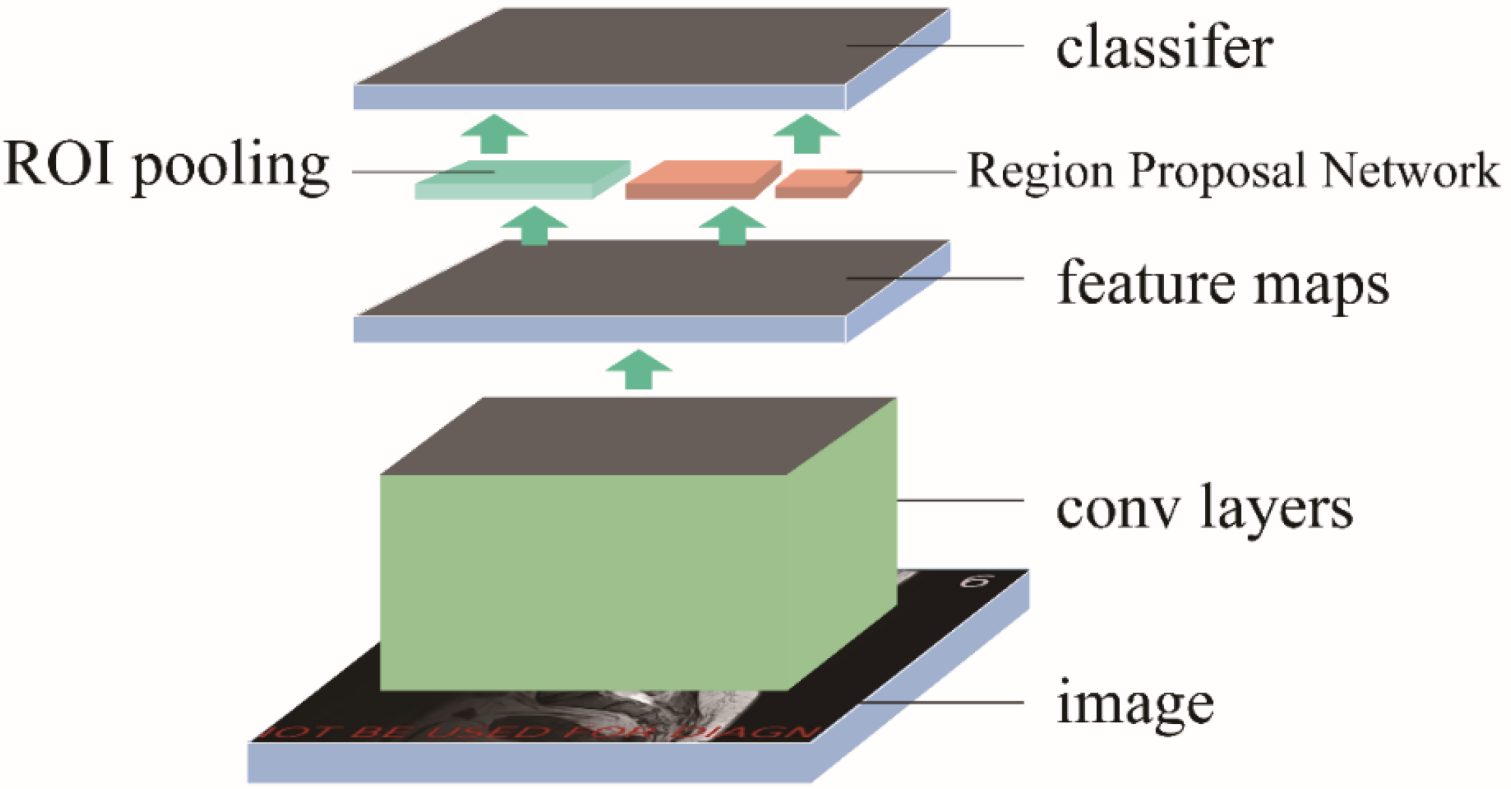
The illustration of Faster R‐CNN. Faster R‐CNN, Faster region‐convolution neural network.

For the subsequent analysis, localization is an essential process that will be helpful in the extraction of regions of interest (ROIs) and help surgeons localize the herniated area efficiently. In this step, the network employed is Faster R‐CNN (Faster region‐convolution neural network), the structure is shown below in Figure 2. Faster R‐CNN is a classic and verified algorithm used widely in industry. It generally uses RPN (region proposal network) to generate ROIs, then uses a classifier to determine which class each region belongs to.^27^, ^28^ Unlike typical applications, our approach does not directly detect discs. Instead, we detect bones between discs because they have more valid features.

**FIGURE 3:**
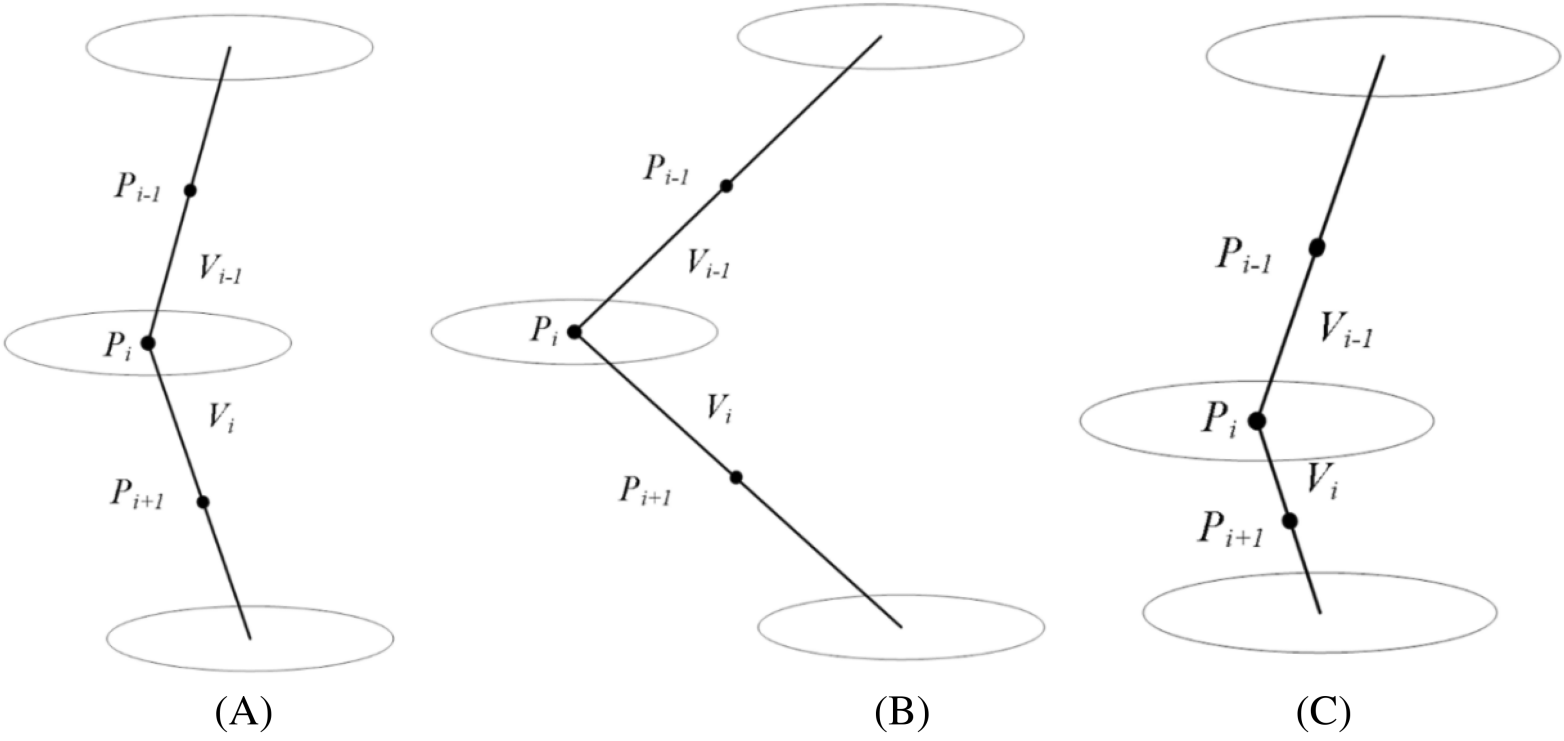
The schematic diagram of distance and angle constraints. (A) Diagram of $P_{i}$ satisfying both distance and angle constraints. (B) Diagram of $P_{i}$ not satisfying the angle constraint. (C) Diagram of $P_{i}$ not satisfying the distance constraint.

Given the limited mobility of the lumbar spine, an optimization algorithm was developed that could replace localization points that deviate from anatomy theoretic points with a well‐positioned one. The algorithm consisted of distance constraint and angle constraint (Figure 3). It functions as a post‐processing step, refining Faster R‐CNN predictions based on anatomical plausibility.

The point *i* was expressed as $P_{i}$. The coordinates of $P_{i}$ was defined as $\;P_{i}$ = ($x_{i},y_{i}$). $V_{i}$ denoted the distance between $P_{i+1}$ and $P_{i}$, defined as:

(1) $$V_{i}=\sqrt{{\left(y_{i+1}-y_{i}\right)}^{2}+{\left(x_{i+1}-x_{i}\right)}^{2}}.$$

The distance constraint was defined as:

(2) $$\lambda_{1}<\frac{V_{i}}{V_{i-1}}<\lambda_{2}.$$

The angle constraint was defined as:

(3) $$\theta_{1}<\arccos\frac{\vec{P_{i}P_{i-1}}\vec{P_{i}P_{i+1}}}{\left|P_{i}P_{i-1}\right|\left|P_{i}P_{i+1}\right|}<\theta_{2}.$$

All of the above‐mentioned constants are predefined experimentally to be $\lambda_{1}=0.6$, $\lambda_{2}=1.25$, $\theta_{1}=140^{{}^{\circ}}$, and $\theta_{2}=180^{{}^{\circ}}$.

If the localization of a point is outside of the agreed range, the localization will be optimized based on the adjacent point. The sine qua non of this optimization algorithm is that a primary neural network can obtain sufficient correct points as a reference.

### 2.4 Diagnosis of LDH

**FIGURE 4:**
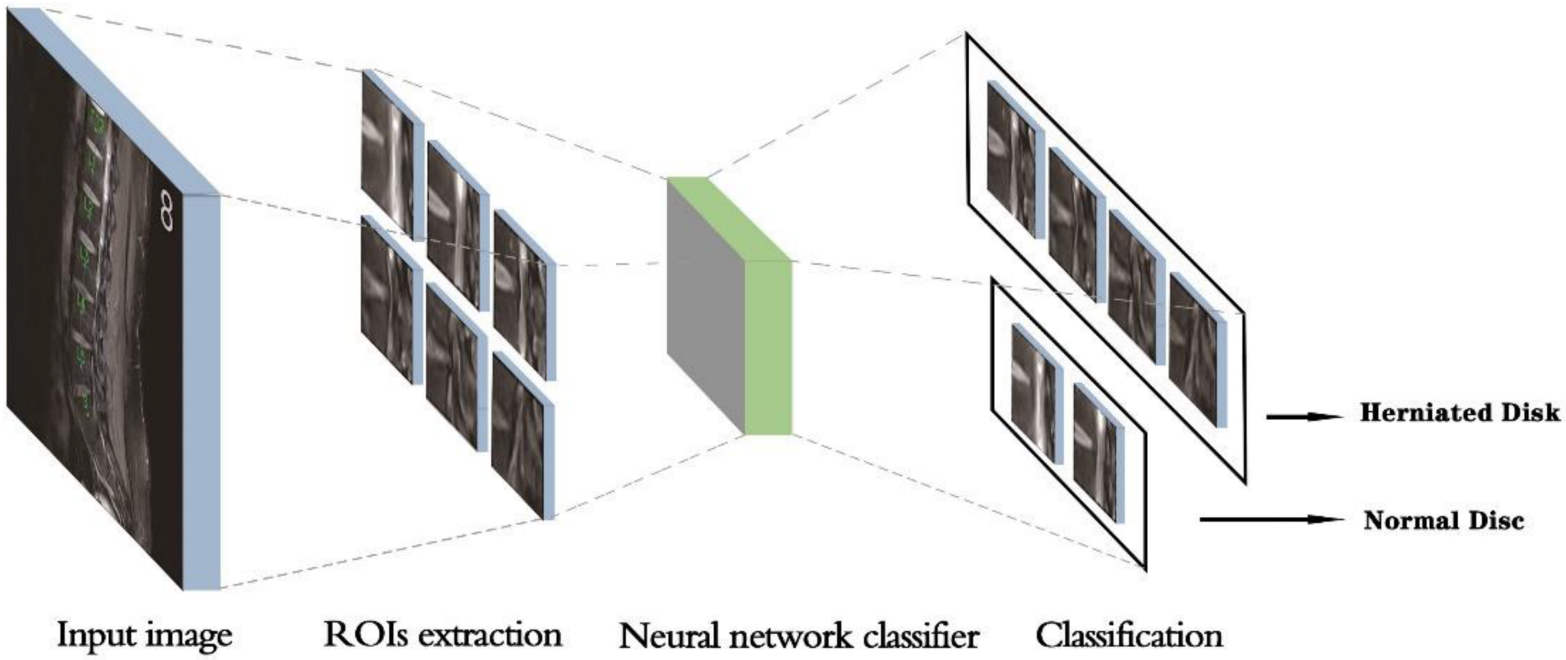
Judging of lumbar disc herniation.

In this step, a binary neural network classifier was adopted. Following the localization of vertebrates, ROIs were generated by cropping the latter half of IVD. Specifically, we connected adjacent points to create a rectangle. One side of this rectangle was defined by the line connecting the localization points of the vertebral bodies, while the other side had a fixed length of 44 pixels. Then, the ROIs would be fed into the neural network algorithm to determine whether each of them represents herniation or not. The structure of the process can be seen in Figure 4.

### 2.5 MSU classification

**FIGURE 5:**
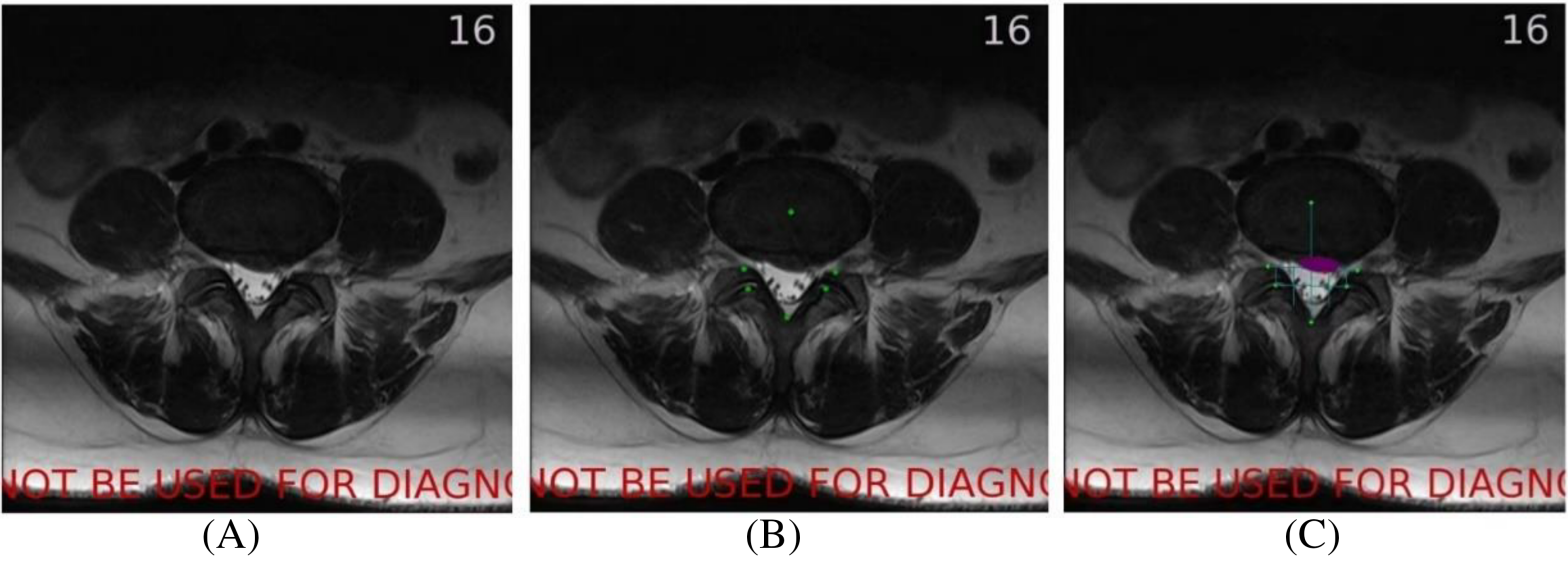
(A) The input image in cross‐section view. (B) Green point: six marker points of MSU classification (center of intervertebral disc, dorsal point of the spinal canal, apex in the ventral side of the superior articular process, apex in the ventral side of the inferior articular process). (C) Red point: apex of herniated area; purple area: herniated area; green line: dividing line for MSU classification.

After the localization of the herniated disc, the cross‐section view of MRI images should be retrieved to conduct MSU classification (Figure 5A). The basic principle of the MSU classification is dividing spinal canal into several regions using several vertical and horizontal lines. The MSU classification could then be determined according to the region in which the herniation lies.^7^

**FIGURE 6:**
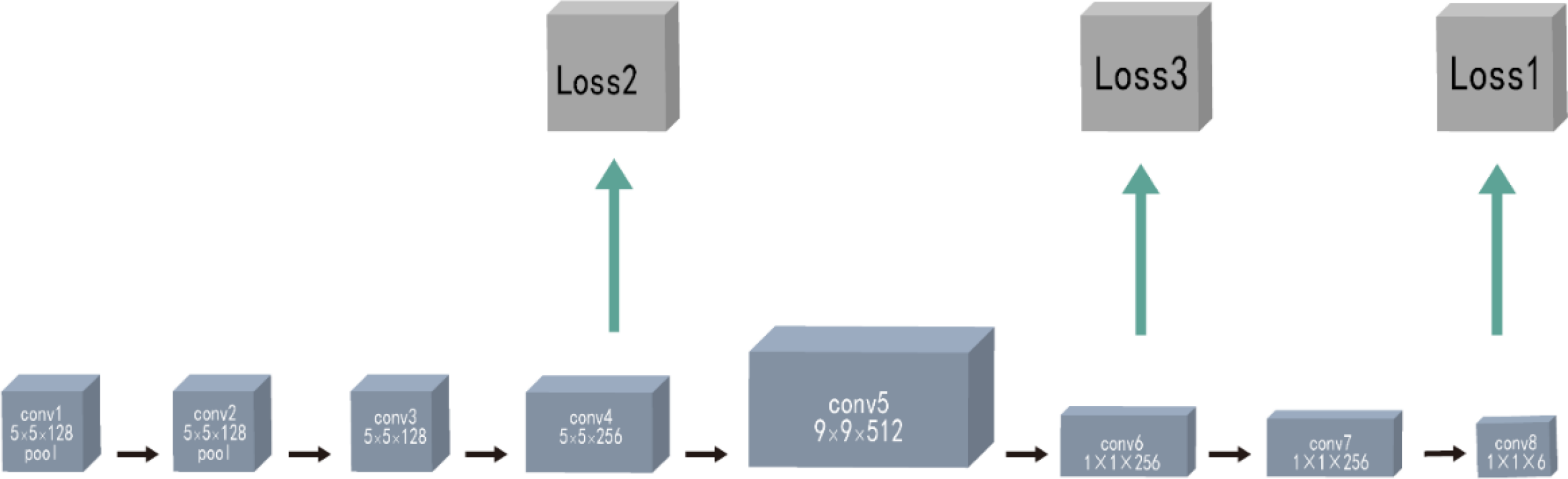
The architecture of key point localization algorithm.

**FIGURE 7:**
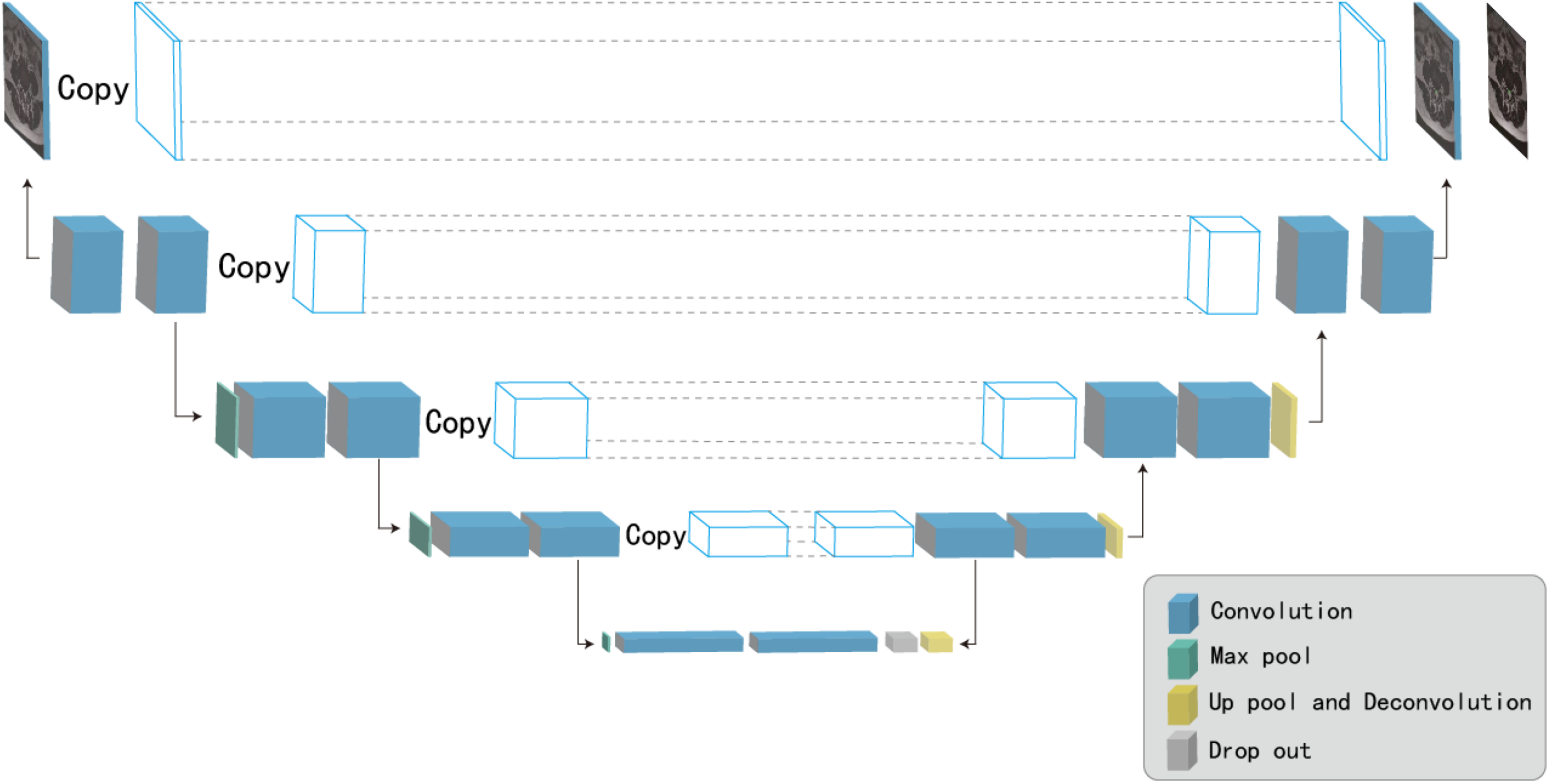
The network structure of U‐Net.

First, a CNN algorithm was used to localize specific marker points in the MSU classification (Figure 5B). Then, a classification map will be generated based on these points. Subsequently, the herniation areas will be segmented out (purple area in Figure 5C). The network structure for segmentation was U‐Net.^29^ Finally, depending on the region in which the apex of the herniated area (red point in Figure 5C) lies, an MSU classification could be assigned (Figure 5C). The network structure of CNN and U‐Net are shown in Figure 6 and Figure 7, respectively.

### 2.6 Pfirrmann grade of disk

After diagnosis and MSU classification, the Pfirrmann grade will be assessed, which is critical to the decision‐making between decompression and interbody fusion.

**FIGURE 8:**
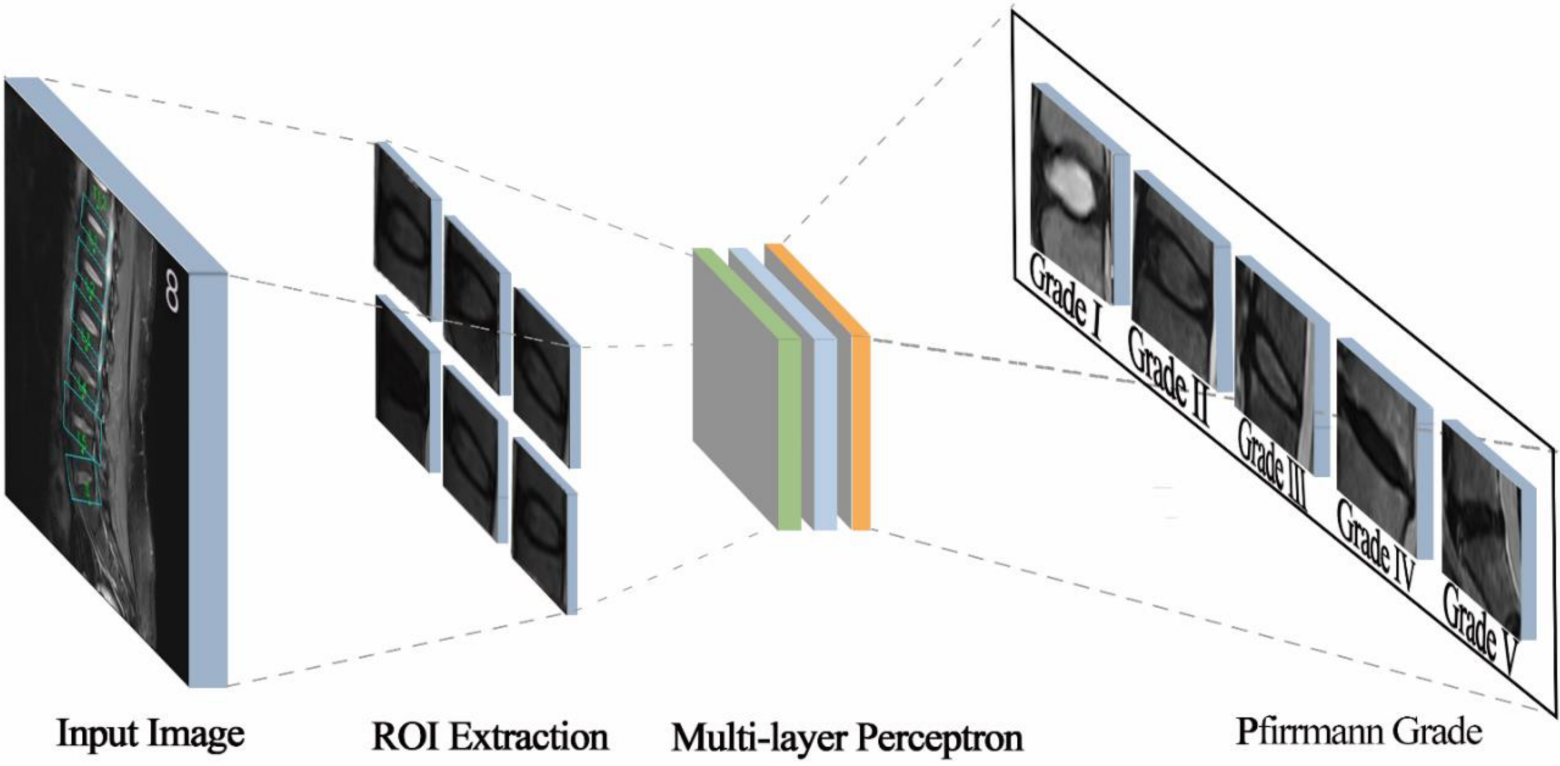
General structure of an MLP‐based predictive model for disc herniation. MLP, multi‐layer perceptron.

The system described bone localization as the first step to tackle the Pfirrmann grade. For each IVD, a bounding rectangle was created whose width and orientation were about the same as the line connecting adjacent bone centers. The cropped rectangle was then fed into an ML model to predict the Pfirrmann grade. (Figure 8).

The prediction model was a multi‐layer perceptron (MLP) classifier whose input feature was carefully designed and tuned.^30^ Specifically, the following image features are computed:

Histogram of pixel intensity (HPI): when degeneration or herniation happens, the IVD will have water loss. As a result, the image will appear darker, and the histogram statistics will reflect this.

Local binary pattern (LBP): LBP is commonly used as a local texture descriptor by comparing each pixel with its surrounding neighbors. The texture reveals the disc pathologies; hence, LBP is a natural choice.

Pyramid Histogram of Oriented Gradients (PHOG): PHOG is the de facto image feature used in many computer vision tasks. PHOG serves as a power shape descriptor by computing the gradient orientation and magnitude.

The above features were normalized and concatenated into a single feature vector, which was then fed into the MLP classifier. And then, the model could be trained by stochastic gradient descent.

The MLP architecture has 572 input neurons and two hidden layers, with 300 neurons in the first layer and 150 in the second; in the output layer, there were 5 neurons representing five class of Pfirrmann grade.

### 2.7 Accuracy evaluation

There were several standards to analyze AI diagnosis results. The sensitivity was used to specify the accuracy of disc localization. For LDH diagnosis and Pfirrmann grade, metrics such as accuracy, specificity, precision and sensitivity need to be considered. For MSU classification, accuracy was used as benchmark. Besides, the accuracy of MSU classification mainly depends on the accuracy of point localization and segmentation, so to further evaluate the MSU classification result, point localization accuracy and segmentation accuracy were also reported. Point localization accuracy is determined using the Euclidean distance. Segmentation accuracy is the mean intersection over union (IOU) and dice similarity coefficient (DSC) between predicted regions and ground‐truth regions.^31^

Utilize specificity, sensitivity, precision, accuracy, DSC, and IOU as metrics for performance evaluation which are defined as:

Specificity=TNFP+TNSensitivity=TPTP+FNPrecision=TPTP+FPAccuracy=TP+TNTP+TN+FP+FNDSC=2TPFP+2TP+FNIOU=TPFP+TP+FN

TP (True Positives): The intersection of ground‐truth and model predictive area.

TN (True Negatives): The area not labeled in ground‐truth and not recognized by the ML model.

FN (False Negatives): The ground‐truth label not recognized by the model.

FP (False Positives): The area predicted by the ML model but not labeled in ground‐truth.

### 2.8 Model validation

To test the decision support system, the performance of 4 in‐training surgeons (S.J.J., L.S.W., F.T., and C.J.Y., all with ≤3 years of experience) was compared with and without ML assistance. They were divided into two groups evenly.

Before interpretation, all surgeons underwent familiarization with the diagnosis of LDH, Pfirrmann grade, and MSU classification.

**FIGURE 9:**
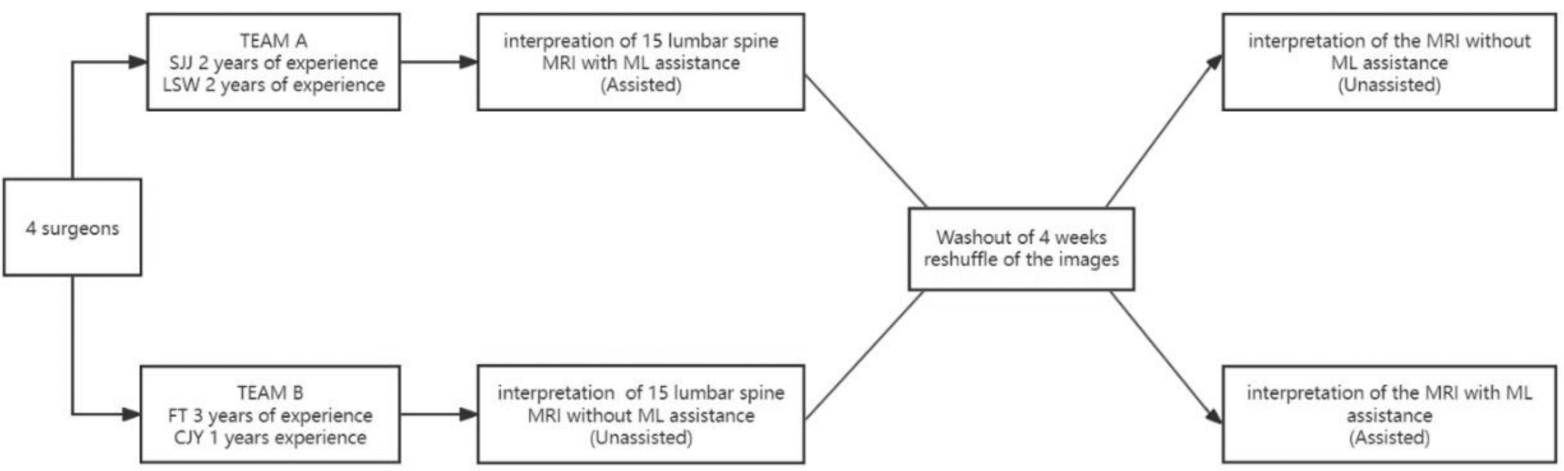
The graphic representation of the crossover study design.

The four surgeons were equally divided into two groups. Initially, Team A conducted the interpretation of 15 lumbar spine MRI scans, encompassing tasks such as diagnosis, Pfirrmann classification, and MSU classification, with the aid of an ML model. Team B undertook the same task, but without ML assistance. Following a washout period of 1 month, the order of 15 lumbar spine MRI was randomly reshuffled to prevent familiarization. Team A then interpreted without model assistance and Team B interpreted the images with model assistance. During the ML‐assisted readings, radiologists were presented with bounding box predictions provided by the ML model. The crossover study design is illustrated in Figure 9.

For this study, the primary outcomes were interpretation accuracy, time taken to interpret each spine MRI, and interrater agreement assessed by Fleiss' kappa. All analyses were performed by J.W.D and D.Z using SPSS (Version26. IBM Corp).

## 3 RESULTS

### 3.1 Data set

This study was based on a patient population from 2017 to 2018 in the General Hospital of Tianjin Medical University. Our team selected the middle three images in sagittal view. A total of 217 patients' MRI scans were collected for evaluation, comprising 3255 lumbar disc images (217 patients * 3 images * 5 discs). The pixel size of the images is 0.2646 mm per pixel. They were divided into two groups: training group for 184 patients (2760 lumbar disc images) and testing group for 33 patients (495 lumbar disc images). MRI scans were mainly obtained from the Department of Radiology. Image scanning was not standardized and may come from different machines. The baseline characteristics of the patients are provided in Table 1.

**TABLE 1:** (A) Baseline of patients, (B) baseline of LDH, (C) baseline of Pfirrmann grade, and (D) baseline of MSU classification.

(A)	
	Mean ± SD
Age (years)	60.4 ± 10.7
BMI (kg/m^2^)	27.1 ± 4.2
Gender, male/female	98/119

(B)		
	Herniated disc	Normal disc
L1‐L2	543	108
L2‐L3	607	44
L3‐L4	626	25
L4‐L5	639	12
L5‐S1	618	33

(C)	
I	130
II	780
III	1094
IV	875
V	376

(D)			
MSU	1	2	3
A	126	140	27
B	144	202	39
C	40	68	0
AB	38	126	18

### 3.2 Localization and diagnosis of LDH

The quality of the localization was defined as sensitivity. The threshold for recall was set as 4 mm after pre‐experimentation. The sensitivity for Faster RCNN is as high as 95.0%, and 99.3% after optimization. As a result of optimization algorithm, the error location was reduced by 86%. There is a mean Euclidean distance of 2.11 mm between ground‐truth and model‐predicted points, with a minimum distance of 1.00 mm and a maximal distance of 5.38 mm.

For the diagnosis of LDH, accuracy is the benchmark. The accuracy of diagnosis is 95.83%, with 97.52% specificity and 94.12% sensitivity.

### 3.3 MSU classification and Pfirrmann grade

For MSU classification, 95.0% of cases were correctly classified (95.6% specificity and 93.8% sensitivity). The system showed a 3‐pixel error on average for key point detection. The segmentation process yielded a mean IoU of 90.4%, with a DSC of 95.0%. The accuracy for the Pfirrmann grade is 83.5%. Moreover, a detailed breakdown of precision and sensitivity across different Pfirrmann grades is presented in Table 2, offering a comprehensive view of the system's performance across varying degrees of severity.

**TABLE 2:** Pfirrmann grade classification performance.

Pfirrmann grade	Precision	Sensitivity
I	68.46%	78.46%
II	83.98%	83.33%
III	85.12%	84.73%
IV	84.37%	83.89%
V	81.77%	81.12%

### 3.4 Model validation/performance evaluation

The data set for validation was chosen randomly from the training and test data set, so this research assumed the patients' baseline characteristics were same as the training data. The ground‐truth label was used as the reference standard. Fifteen patients' lumbar spine MRIs were randomly chosen as the validation data set (75 discs in sagittal view for diagnosis and Pfirrmann grade, 60 herniated discs in horizontal view for MSU classification).

For the ML‐assisted group, the accuracy of the diagnosis or classification improved by 48.36% on average, the interpretation time had a mean 35.77% reduction. There was an increase in interrater agreement for diagnosis (Fleiss' kappa went from 0.484 to 0.741), as well as for Pfirrmann grade (from 0.330 to 0.669) (Table 3).

**TABLE 3:** Comparison of accuracy, interpretation time, and interrater agreement with and without ML model assistance.

	Diagnosis	Pfirrmann grade	MSU classification
Accuracy (unassisted)^a^	61.67 ± 8.76%	53.67 ± 19.15%	46.77 ± 8.85%
Accuracy (assisted)^a^	77.00 ± 7.39%	71.00 ± 17.80%	87.90 ± 2.68%
Time consumed (unassisted) (s)^a^	496.5 ± 141.7	506.95 ± 158.8	1216.5 ± 253.3
Time consumed (assisted) (s)^a^	321.8 ± 54.0	373.05 ± 74.44	660.5 ± 139.0
Interrater agreement (unassisted)	0.484 ± 0.047	0.330 ± 0.028	‐
Interrater agreement (assisted)	0.741 ± 0.047	0.669 ± 0.029	‐
Accuracy improved	24.86%	32.30%	87.93%
Time reduction	35.19%	26.41%	45.70%

Abbreviation: ML, machine learning.

^a^ Data are mean ± std.

## 4 DISCUSSION

In this work, an automatic clinical decision support system based on ML was proposed, and a clinical pathway was presented with three steps: (1) diagnosis, (2) MSU classification, and (3) Pfirrmann grade. The performance evaluation of the system indicated that DSS could improve the performance of in‐training surgeons by a substantial margin.

According to the data, the localization sensitivity in this study was 99.5%. The accuracy of Pfirrmann grade in this study was 83.5%. The intrarater agreement presented by Pfirrmann et al. and Niemeyer et al. is 0.745 and 0.880, which are always seen as the rooftop for ML models.^10^, ^20^ The accuracy of MSU classification reached 95.0% with a 95.2% DSC in the segmentation of herniated area. Compared to state‐of‐the‐art research, the localization and segmentation algorithm shows considerable superiority.^19^, ^21^, ^32^, ^33^, ^34^

MLP is on par with CNN^22^ in Pfirrmann grade with a relatively small data set and primitive architecture. The result is better than expected. It concluded that the traditional computer vision method could produce a better result with carefully selected descriptors for the mission with clear descriptions.

Compared with the unassisted surgeon, the ML‐assisted group showed both higher accuracy rate and shorter time consumption. The accuracy improvement of the ML‐assisted group may be explained by the inexperience of the participating surgeon and the ambiguity of some intractable cases. However, the validation process has limitations: first, the study and the data set were conducted in a single center which may introduce bias; second, some of the time saving in this study may be due to the automated report generation but not entirely due to faster interpretation using the ML model.

It has been observed that the accuracy of in‐training surgeons is below the anticipated level in the validation process. This phenomenon can be attributed to the ground‐truth labeling. The research team deliberately configured the ground‐truth labeling to have high sensitivity and low specificity, which resulted in certain cases that are conventionally identified as bulging discs being classified as herniated discs. The majority of mislabeling by surgeons can be traced back to the inherent bias within the ground‐truth data.

The MSU classification is a simple and reliable method to objectively describe the size and location of the herniated area.^7^, ^8^, ^9^ The herniation size was classified as 1‐2‐3, and the location was classified as A‐B‐C. It is widely acknowledged that MSU‐1 is suitable for non‐surgical treatment and for MSU‐2,3 surgical treatment should be in consideration.^7^, ^8^, ^9^ In the case of MSU‐A, microendoscopic discectomy or posterior approach surgery could be performed. MSU‐B, C will suit percutaneous transforaminal endoscopic discectomy or Oblique Lateral Interbody Fusion which are lateral approaches.

The most used surgery for LDH is decompression. The aim of lumbar decompression surgery is to relieve the pressure on the spinal cord or nerve roots while maintaining as much of the strength and flexibility of the spine as possible.^35^, ^36^ Nevertheless, as with other surgeries, there are also both risks and benefits. It produces side effects such as recurrence of LDH, insufficient decompression, and subsequent spondylolisthesis caused by spinal instability.^37^, ^38^ According to various research, the Pfirrmann grading scale has a prognostic value for the side effect. Pfirrmann grade >3 often suggests reoperation^39^, ^40^; therefore, lumbar interbody fusion should be in consideration.^41^

The advantages of this method: 1. Accurate localization and segmentation of herniated discs. 2. Treatment suggestion according to diagnosis as well as Pfirrmann grade and MSU classification. 3. Clinical route with objective criteria which could reduce observer bias. 4. Fully automatic. 5. The data as MRI image is the gold standard for the diagnosis of LDH.

The limitations of this study: 1. All images come from a single center, and the manual delineation and evaluation were made by several spine surgeons from the same institution. The consistency of the labeling process may make it easy for the model training because of less confusion. However, this may result in systemic prejudice. Further external validation of the accuracy of the system will be useful to determine its generalizability. 2. The model is only based on images. Further integration of patient's symptoms and other information through NLP (natural language process) will be useful for clinical decision‐making.

## 5 CONCLUSION

Automated accurate and reliable MRI‐based decision support system for LDH establishes an efficient diagnostic method and an objective criterion for treatment suggestion. Reducing time and observer bias will alleviate the burden of clinical work. This study provides the foundation for further research in different diseases and clinical applications.

## CONFLICT OF INTEREST STATEMENT

The authors declare no conflicts of interest.
